# Feasibility of using Virtual Reality in Patients with Panic Disorder: An Experimental Crossover Study Protocol

**DOI:** 10.2174/0117450179487637260905052225

**Published:** 2026-09-08

**Authors:** Sergio Machado, Rafael Ferreira-Garcia, Lucio Lage Gonçalves, José Carlos Appolinario, Antonio E. Nardi

**Affiliations:** 1 Virtual Reality in Mental Health Laboratory (RVSM) - Institute of Psychiatry (IPUB), Federal University of Rio de Janeiro (UFRJ), Rio de Janeiro, Brazil; 2 LABPR – Panic and Respiration Laboratory (LABPR) - Institute of Psychiatry (IPUB), Federal University of Rio de Janeiro (UFRJ), Rio de Janeiro, Brazil; 3 Obesity and Eating Disorder Group (GOTA) - Institute of Psychiatry (IPUB), Federal University of Rio de Janeiro (UFRJ), Rio de Janeiro, Brazil; 4 Center of Neuroscience, Neurodiversity Institute, Queimados- RJ , Brazil

**Keywords:** Anxiety, Agoraphobia, Cognitive-Behavioral Therapy, Panic, Protocol, Virtual reality

## Abstract

**Introduction:**

Virtual reality (VR) is a promising tool for diagnosis and therapy in Panic Disorder (PD), allowing controlled exposure to anxiety-provoking situations and real-time assessment of behaviors and physiological responses. Studies indicate its effectiveness in reducing symptoms and its therapeutic applicability. However, there is a lack of robust data on its specific impact on heart rate variability (HRV), sense of presence, safety, and acceptability in patients with PD and agoraphobia. Therefore, further research is warranted to evaluate these aspects, aiming for a broader understanding and optimization of this technology in mental health.

**Objective:**

Thus, this experimental crossover study protocol aims to evaluate the sense of presence, method safety, acceptability, psychological (*i.e.*, anxiety), and physiological changes (*i.e.*, respiratory and heart rate, HRV, tidal volume and respiratory minute volume, and level of skin conductance). In addition, correlate the psychological measures with the physiological ones.

**Methodology:**

This is an experimental crossover study protocol, following a within-subject crossover study design, with independent groups (between-subjects), in which 25 PD patients and 25 controls will be randomly allocated, through block randomization to an experimental group (PD patients) and a control group (healthy individuals) where they will go through the following sequences: (1) initial exposure to 2D video, using projection glasses, followed by the same scene in VR with the Oculus 2 headset; or (2) initial exposure in VR, followed by the 2D video. VR offers an ecological and multimodal method for assessment, aligned with initiatives such as RDoC. The protocol, structured in modules that reflect components of Cognitive-Behavioral Therapy, aims to test the system's ability to respond to clinically relevant anxiety in a safe and acceptable manner.

**Results:**

The results of the trial will be published in international peer-reviewed journals and will be disseminated at international meetings and congresses.

**Discussion:**

Potential contributions include a more objective and standardized assessment, useful for personalizing interventions. Limitations such as sample size and ecological validity are acknowledged.

**Conclusion:**

In conclusion, the protocol represents a methodological advancement. The results of this predictive study will be crucial for refining the technology and informing future clinical trials, with the potential to improve diagnostic accuracy and optimize personalized therapeutic strategies for PD.

## 1 INTRODUCTION

Panic Disorder (PD) is a psychiatric condition that many people live with. It generates sudden and unex-pected panic attacks, and constant worry about when the next attack might happen [1]. Conventional approaches to assessment and treatment frequently depend on retrospective self-report measures or real-life exposure exercises, strategies that are often logistically demanding, resource-intensive, and hard to implement consistently [2]. Virtual Reality (VR) has become a powerful new tool in clinical psychology, allowing researchers and clinicians to create immersive environments that closely resemble everyday situations [1, 3, 4]. These environments allow for real-time interactions with virtual entities and controlled exposure to feared stimuli [5, 6].

A growing body of literature has examined the psychophysiological responses related to anxiety during VR exposure [5, 7]. Investigations have demonstrated that VR scenarios replicating agoraphobic contexts, such as crowded public areas, confined spaces, and public transit, are capable of consistently eliciting anxiety symptoms, elevating heart rate, and diminishing heart rate variability (HRV) in individuals with PD and agoraphobia relative to healthy controls [8, 9]. With respect to sense of presence, evidence indicates that greater levels of immersion (via VR headsets) yield a more robust sense of presence, which in turn is associated with heightened physiological arousal when contrasted with less immersive platforms like 2D displays [5]. Nevertheless, the relationship between presence and clinical response in PD remains insufficiently characterized, with meta-analytic evidence indicating only a modest association (r = 0.27) between self-reported presence and anxiety during VR exposure [10]. Furthermore, comparatively few investigations have conducted head-to-head comparisons of VR and 2D modalities using a unified experimental protocol [11, 12], and the existing evidence suggests that VR may not consistently outperform 2D delivery in eliciting emotional or physiological responses [13].

Data on the safety profile and acceptability of VR in PD remain limited to early-stage investigation. Shin *et al.* [14] demonstrated that VR-based exposure was well tolerated, with minimal dropout due to cybersickness, and patients reported high satisfaction. More recently, studies have emphasized the importance of assessing cybersickness systematically, as motion sickness symptoms may confound physiological measurements and limit clinical applicability [15, 16]. Regarding HRV, current evidence suggests that patients with PD exhibit reduced vagal tone (lower HRV) at rest and in response to threat cues [17], and that VR exposure may amplify this autonomic inflexibility [18]. Nevertheless, the literature remains sparse on how the immersive modality (VR *vs.* 2D) differentially affects HRV parameters in PD, which is a gap the present protocol aims to address.

The use of VR reduces human resources and costs, offering advantages in terms of sustainability, acceptability, and safety of the method [7, 18]. Accordingly, this protocol has three main objectives. First, to evaluate the sense of presence (*i.e.*, the subjective experience of being there in the virtual environment) by comparing immersive VR to 2D video, and to assess how presence relates to anxiety responses in patients with PD. Second, to establish the discriminant validity of the VR paradigm by confirming that the subway environments elicit significantly greater anxiety responses in PD patients compared to healthy controls, thereby demonstrating that the paradigm is specifically sensitive to PD-related threat processing. Third, to characterize the physiological signature of PD by comparing HRV, skin conductance, and respiratory patterns between groups during controlled exposure, contributing to the identification of potential physiological biomarkers for PD. To support these objectives, a healthy control group was included to serve as a reference for both the subjective and physiological measures.

Accordingly, we propose the following hypotheses. The first one related to sense of presence: Immersive VR exposure will produce significantly higher presence scores (IPQ) compared to 2D video in both groups, and presence will be positively correlated with anxiety intensity (SUDS) and physiological reactivity (heart rate, skin conductance) during VR exposure. The second one is associated with discriminant validity: PD patients will show significantly higher subjective distress (SUDS) and greater physiological reactivity (increased heart rate, reduced HRV, increased skin conductance) during VR exposure compared to healthy controls, confirming that the paradigm is specifically sensitive to PD-related threat processing. The third one is connected to physiological signature: PD patients will exhibit a distinct physiological profile during VR exposure relative to controls, characterized by lower HRV (reduced RMSSD and HF power), elevated skin conductance levels (SCL), and greater respiratory irregularity (respiratory rate and tidal volume). These physiological parameters will be significant predictors of PD symptom severity as measured by the PDSS.

## 2 METHODS

### 2.1 Study Design

This is an experimental crossover study protocol following a within-subject crossover design with independent groups. Participants of both the genders will be allocated into two predefined groups based on clinical status: (1) patients with a primary diagnosis of PD (with or without agoraphobia) and (2) healthy controls matched by age, sex, and educational level. Randomization applies exclusively to the order of exposure conditions within each group: participants in both groups are randomly assigned (via block randomization using www.randomization.org) to either Sequence A (2D video first, followed by VR) or Sequence B (VR first, followed by 2D video). Thus, group allocation (PD *vs.* Control) is determined by clinical status, while condition order (2D→VR *vs.* VR→2D) is randomized and counterbalanced. To address potential carryover effects, a 15-minute washout period is interspersed between the two exposure conditions, and the effect of sequence order will be tested as a between-subjects factor in the statistical models.

### 2.2 Sample

The sample will consist of 50 participants, divided into two groups: 25 PD patients (with or without agoraphobia) and 25 healthy controls. The sample size was calculated a priori using G*Power software (version 3.1) [ 19 ]. For a mixed-design ANOVA with two groups (PD patients *vs.* controls) and two within-subject conditions (VR *vs.* 2D video), aiming to detect a medium-to-large effect size (Cohen's f = 0.25, equivalent to partial η ^2^ = 0.06), with a power of 0.80 (1 − β), a significance level of α = 0.05 (two-tailed), a correlation of 0.50 between repeated measures, and a nonsphericity correction ε = 1, the required total sample size is N = 44 (22 per group). To account for potential dropouts and missing data (approximately 12%), the final sample size was set at N = 50 (25 per group), consistent with previous feasibility studies in similar populations. This sample size is adequate to detect main effects and interaction effects of medium to large magnitude, which is appropriate for a feasibility study aiming to establish preliminary evidence.

The sample of PD will be recruited from the Institute of Psychiatry (IPUB/UFRJ). Inclusion criteria for the PD group: (1) primary diagnosis of PD made by an experienced psychiatrist according to the Diagnostic and Statistical Manual of Mental Disorders, Fifth Edition, Text Revision (DSM-5-TR); (2) have experienced at least two panic attacks in the 30 days prior to the visit; (3) age 18-65 years, (4) have a score ≥ 8 on the Panic Disorder Severity Scale (PDSS) [ 20 ] and (5) participate voluntarily with signed informed consent. Exclusion criteria for PD patients will be: (1) current psychiatric comorbidities; (2) neurological impairment that may interfere with the Virtual Reality (VR) experience; (3) current substance use disorder (except nicotine), cardiovascular disease, respiratory disease, epilepsy, vestibular disorders, and pregnancy. (4) medications categorized as: SSRIs, SNRIs, benzodiazepines, tricyclic antidepressants, and others. At screening, a structured clinical interview will document the type, dosage, duration of use, and time since last dose for each psychotropic medication. Specifically, benzodiazepine use within 12 hours before the experimental session will be an additional exclusion criterion, given their acute suppressive effect on HRV and potential to mask anxiety responses. Thus, medication status will be included as a covariate in sensitivity analyses to assess its potential confounding effect on physiological outcomes.

The control group will be similarly evaluated, and participants will be individually matched to PD patients on age (± 3 years), sex, years of education (± 2 years), technological familiarity, and prior exposure to VR. Inclusion criteria for controls will be as follows: (1) age 18-65 years and (2) voluntary participation with signed informed consent. Exclusion criteria for healthy controls will be as follows: (1) history of or current mental disorders; (2) neurological impairment that may interfere with the Virtual Reality (VR) experience; (3) current substance use disorder (except nicotine), cardiovascular disease, respiratory disease, epilepsy, vestibular disorders, and pregnancy.

Each patient will be informed about all experimental procedures and will sign a written consent form, and the experiment was approved by the ethics committee of the Institute of Psychiatry of the Federal University of Rio de Janeiro (IPUB-UFRJ) (CAAE 74103623.0.0000.5263).

### 2.3 Experimental Procedures

After screening, eligible participants will sign the Informed Consent Form (ICF). They will then answer a questionnaire developed by our team to collect sociodemo-graphic information and contact details, as well as answer questions related to subway use, such as previous experiences of discomfort or avoidance. Two instruments validated for Brazilian Portuguese will also be applied: the Big Five Inventory (BFI-44), to assess personality traits [21], and the Computer Proficiency Questionnaire (CPQ), to assess technological proficiency [22].

The study is an experimental crossover protocol, with an intra-subject and independent-groups crossover design, an experimental group (patients with PD) and a control group (healthy individuals).

The experimental protocol consists of a single session lasting approximately 90 minutes. After initial screening, participants undergo two exposure conditions (2D and VR) in a randomized order: (1) initial exposure to 2D video using projection glasses, followed by the same scene in VR with the Oculus Quest 2 device; or (2) initial exposure in VR, followed by the 2D video. The virtual scenes will represent three subway environments: the turnstile, the platform, and the train car. A team member will remain close to the participant throughout the session to provide instructions on how to use the equipment, explain the scenarios, ensure physical safety, and guide interaction with the virtual environment, including when to respond to the Subjective Units of Discomfort Scale (SUDS) [23].

Outcome measures will be collected at specific times. Immediately before and after each scene [(1) the entrance turnstile for 2 minutes; (2) the boarding platform for 2 minutes; and (3) the interior of a train car for 5 minutes], in both 2D and VR conditions, the participant will complete the SUDS to report their subjective level of discomfort. At the end of each complete condition (which includes the three scenes), the Igroup Presence Questionnaire (IPQ) [24] will be applied to measure the feeling of presence, and the Simulator Sickness Questionnaire (SSQ) [25] to assess symptoms of motion sickness (simulator sickness).

In parallel, physiological data on Heart Rate Variability (HRV) will be recorded with the Polar V800 monitor, with a sampling rate of 1000 Hz. HRV will be measured at rest, before and after each complete condition (the three scenes), with the participant seated in a comfortable chair in an air-conditioned room. Each recording will last 10 minutes (5 minutes for stabilization and 5 minutes of effective collection after stabilization). Data relating to the 5 minutes after stabilization will be extracted and analyzed by Kubios HRV software, after visual inspection and noise correction [26].

In addition, qualitative data will be collected through video recording of participants' behaviors, interactions, and verbalizations during the exposures. After both conditions are completed, a semi-structured interview will be conducted to explore aspects such as usability, interaction with the virtual environment, discomfort, details of the scenario design, sense of presence, and suggestions from participants.

### 2.4 Blinding

Due to the nature of the intervention, participants and researchers delivering the VR or 2D video sessions cannot be blinded to the exposure condition. However, outcome assessors responsible for analyzing physiological data (HRV, skin conductance, respiratory parameters) will be blinded to group allocation (PD patients *vs.* controls). The data will be coded with numerical identifiers, and the assessors will have no access to the allocation list during analysis. Statistical analyses will also be performed by a researcher blinded to group assignment. This single-blind design aims to minimize detection bias in the physiological and psychological outcome measurements.

### 2.5 Outcomes

Primary Feasibility Outcomes: (1) Recruitment rate (≥80% of target), (2) Completion rate (≥80% completing both conditions), (3) Data quality (≥90% usable physiological signal), (4) Acceptability (mean SSQ < 15), (5) Sense of presence (IPQ scores comparable to clinical norms). The protocol is considered feasible if ≥4 of 5 criteria are met.

Secondary Outcomes: (a) Anxiety trajectory (SUDS scores across scenes and conditions), (b) Physiological reactivity (HRV indices: RMSSD, LF/HF, SDNN; skin conductance; respiratory parameters: rate, tidal volume, irregularity), (c) Correlation between psychological measures (SUDS, IPQ) and physiological measures (ΔHRV, Δskin conductance), (d) Qualitative data from the semi-structured interview regarding usability and acceptability.

### 2.6 Virtual Reality Exposure Task

The VR task is a three-dimensional computer animation developed by TriptyqueLAB (Rio de Janeiro, RJ, Brazil) (www.triptyquelab.com). Each subject in each group will go through the following conditions: (1) initial exposure to the 2D video, using projection glasses, followed by the same scene in VR with the Oculus Quest 2 headset; and (2) initial exposure in VR, followed by the 2D video.

In the first level, called the 3D condition, the participant uses an Oculus Quest 2 headset to actively experience and navigate a real-scale virtual environment that simulates a subway, composed of three sequential scenes: (1) the entrance turnstile (2 minutes), (2) the boarding platform (2 minutes), and (3) the interior of a train car (5 minutes). In the second level, the 2D condition, the participant passively watches a video on a TV screen, which presents a first-person filming of the same virtual environments as the three scenes. The order in which these two conditions are presented (RV first or 2D first) is also manipulated through a block randomization procedure.

### 2.7 Psychological Instruments

Big Five Inventory (BFI) assesses the basic personality dimensions of participants, in its adapted and validated version for Brazilian Portuguese [21]. The BFI is a widely recognized self-report instrument, based on the Five-Factor Model of Personality, which assesses five broad and relatively independent dimensions: Extraversion, Agreeableness, Conscientiousness, Neuroticism, and Openness to Experience. The version used consists of 44 items, in which participants indicate how well each statement describes them on a 5-point Likert scale, ranging from 1 (Strongly Disagree) to 5 (Strongly Agree). Scores for each factor are calculated by averaging the responses to the corresponding items, after inverting the scores of negatively formulated items. The instrument demonstrates good psychometric properties in the Brazilian population, with internal consistency coefficients (Cronbach's alpha) ranging from 0.62 to 0.80 for the five scales. In the present study, the BFI was applied in a single session, before the start of the intervention protocol, with the aim of characterizing the personality profile of the sample and verifying the initial equivalence between the experimental groups.

To comprehensively assess the participants' proficiency and comfort in using digital technologies, the Portuguese version of the Computer Proficiency Questionnaire (CPQ), adapted and validated for the Brazilian population [22], was applied. The version used consists of 33 items. For each item, participants rate their own ability to perform a specific task (*e.g.*, “Send an email with an attached file,” “Set up a new mobile app”) on a 5-point Likert scale, ranging from 1 (“I am not able to do this”) to 5 (“I am very good at this”). The option “I have never tried/I don't know what it is” is also provided, scored as 1. The total technological proficiency score is calculated by averaging all valid responses (theoretical range: 1 to 5), with higher scores indicating greater perceived proficiency. The instrument has excellent psychometric properties, with high internal consistency (Cronbach's alpha > 0.90 for the total score) and demonstrated convergent validity.

SUDS is a semi-quantitative assessment method with scores ranging from 0 (no anxiety) to 10 (maximum anxiety) to measure the subjective intensity of distress or suffering currently experienced by an individual [23].

IPQ is a 13-item scale used to measure the sense of presence in a virtual environment [24]. People who are present in a virtual environment have the experience of actually being in the virtual place; they focus their attention on that environment, and they experience it as something real. The IPQ has four subscales: general presence, spatial presence, engagement, and experienced realism.

SSQ used the version translated into Brazilian Portuguese by Carvalho *et al.* [25], which assesses 16 symptoms grouped into 3 factors: oculomotor, nausea, and disorientation. Each symptom is assessed using a four-point scale (0=none, 1=mild, 2=moderate, and 3=severe). In addition to the overall scores for each factor, a total score can be calculated.

### 2.8 Recording and Analysis of Physiological Data

The MP100 with AcqKnowledge software, a pneumotach transducer, ECG, EKG, and skin conductance electrodes from Biopac Systems, Inc. (Goleta, California, USA) (www.biopac.com) will be used. In the skin conductance analysis, the parameters used will be skin conductance (SCL) and electrodermal response magnitude (ERM), which is the average amplitude of electrodermal responses over a given period. The respiratory parameters will be respiratory rate, tidal volume, and minute respiratory volume (MRV). To facilitate comparisons between subjects, the skin conductance level (SCL) will be standardized using a percentage of each subject’s baseline measurements (first gray screen). To measure irregularities in respiratory rate and tidal volume, the von Neumann statistic was used in a manner similar to that described by Abelson *et al.* [27]. This statistic is the sum of the squared differences between successive breaths divided by the number of differences summed. Using this statistical tool, two new variables will be created: respiratory rate (RR) irregularity and tidal volume (TV) irregularity. The means of all these physiological variables will be calculated at twelve 20-second epochs. HRV indices will be analyzed using Kubios™ HRV software (Biomedical Signal Analysis Group, Department of Applied Physics, University of Kuopio, Kuopio, Finland) considering a 5-minute post-stabilization period [26]. The data will be visually inspected to identify noise in the signals (≤2%), which will be manually removed, with the adjacent RR interval values interpolated (filter power < average [27]. The dependent variables will be analyzed in the frequency domain (low frequency [LF], high frequency [HF], and sympathovagal balance [LF/HF]) and time domain (beat-to-beat intervals [RR], standard deviation of the mean of the qualified NN interval [SDNN], proportion of successive NN intervals with a difference greater than 50 ms [pNN50], and mean squared difference of successive normal RR intervals [rMSSD] will also be measured [27].

### 2.9 Statistical Analysis

All statistical analyses will be performed using SPSS (version 25) and R (version 4.3), with a two-tailed significance level set at α = 0.05. Confidence intervals will be reported at 95%. Normality will be assessed using the Shapiro-Wilk test and visual inspection of Q-Q plots; homoscedasticity will be evaluated with Levene's test. Where parametric assumptions are violated, appropriate transformations or non-parametric alternatives will be employed. Effect sizes will be reported for all primary analyses (partial η ^2^ , Cohen's d, and standardized regression coefficients).

To test Hypothesis 1, a mixed ANOVA will be conducted with Condition (VR *vs.* 2D video) as the within-subject factor and Group (PD patients *vs.* healthy controls) as the between-subject factor, using the total IPQ score as the dependent variable. Pearson (or Spearman) correlation coefficients will then be calculated between IPQ scores and SUDS ratings, as well as between IPQ scores and physiological reactivity indices (Δ heart rate, Δ skin conductance level) during VR exposure. A regression analysis with interaction terms will also be performed to test whether the relationship between presence and physiological arousal is moderated by group membership.

To test Hypothesis 2, a mixed ANOVA will be applied to SUDS scores, with Condition (VR *vs.* 2D) as the within-subject factor and Group (PD *vs.* control) as the between-subject factor. Pairwise comparisons with Bonferroni correction will follow significant interactions. The same 2 × 2 mixed ANOVA structure will be applied to physiological outcomes, heart rate, HRV indices (RMSSD, LF/HF), and skin conductance level (SCL), to test whether PD patients exhibit greater physiological reactivity specifically during VR exposure relative to controls. A significant Group × Condition interaction is expected for each outcome.

To test Hypothesis 3, we will first compare groups on resting-state HRV indices (RMSSD, HF power, LF/HF ratio) using independent t-tests. Then, for each physiological parameter during VR exposure (HRV, SCL, respiratory rate, tidal volume irregularity), a mixed ANOVA (Group × Condition) will be conducted as described above. To determine whether these physiological parameters predict PD symptom severity, a multiple linear regression will be performed with PDSS score as the dependent variable and the physiological indices (ΔHRV, ΔSCL, respiratory irregularity) entered as predictors, controlling for age, sex, and medication status.

To control for Type I error across the multiple physiological outcomes, a Holm-Bonferroni correction will be applied within each family of tests. Sensitivity analyses will be conducted including medication status (categorized) and sequence order (2D→VR *vs.* VR→2D) as covariates in the main models to assess their potential confounding effects. Where appropriate, multilevel linear mixed-effects models will be used to account for the nested structure of repeated physiological measurements across scenes and conditions.

## 3 RESULTS AND DISCUSSION

In this context, the development of innovative and ecologically valid assessment methods is a priority. VR emerges as a promising tool, as it allows the patient to be immersed in simulated, controlled, and safe environments that replicate anxiety-provoking situations of daily life [3-5]. This technology allows for the simultaneous and real-time collection of multiple data, integrating subjective measures (self-reported anxiety), behavioral measures (*e.g.*, time spent in the situation), and physiological measures (such as HRV) [5]. This multimodal approach is in line with the Research Domain Criteria (RDoC) initiative, which advocates for understanding mental disorders through biobehavioral domains and constructs that transcend traditional diagnostic categories [28].

This experimental crossover study protocol aims to investigate the feasibility of a VR-based assessment system for patients with PD (VR-PD). The protocol reflects an important component of Cognitive-Behavioral Therapy (CBT) for anxiety: *in vivo* exposure [29, 30] within subway environments. Assessing the feasibility of this system is a fundamental and preliminary step to testing its effectiveness as a therapeutic tool. This assessment encompasses patient acceptability (tolerability, cyber-nausea), the practicality of implementation in clinical settings, the system's ability to induce clinically relevant anxiety responses, and the initial validity of its measures.

The feasibility of VR-PD is suggested by preliminary studies that used similar virtual environments, which demonstrated good acceptability and the ability to induce anxiety in a significant, yet safe, way in patients with PD [7, 18]. Immersion in progressively more crowded subway environments is expected to elicit anxiety responses that are measurable and distinguishable between patients and healthy controls [1, 8, 9]. Similarly, it will be possible to assess, respectively, the capacity for emotional regulation [31] and sensitivity to bodily sensations [32, 33], which are central aspects in the pathophysiology of TP [34].

The main potential contribution of this protocol lies in the possibility of offering a more objective, standardized, and data-rich assessment of each patient's anxiety profile. By capturing not only what the patient reports, but also how their body reacts in real time to specific stimuli, VR-PD can assist in identifying response subtypes, objectively monitoring progression throughout treatment, and personalizing exposure interventions. Furthermore, by reducing the reliance on intensive human resources for creating *in vivo* exposure situations, VR can become a sustainable and scalable tool for mental health services [5].

This protocol, however, is subject to limitations that will be considered in the interpretation of future results. The planned sample size, while adequate for a feasibility study, may limit the generalizability of the findings. The potential influence of psychotropic medications on physiological responses will be an important variable to control. In addition, ecological validity, while superior to traditional laboratory methods, is still a simulation, and the transfer of findings to the real world will require further investigation.

## CONCLUSION

In conclusion, the implementation of this VR-based assessment protocol for PD represents a methodological advancement aligned with contemporary needs for precision and multimodality in psychiatry. The results of this feasibility study will be crucial for refining the technology, establishing parameters for safe and effective use, and providing a basis for future randomized clinical trials testing the efficacy of VR-PD as a complementary assessment and intervention tool. Successful validation of this system may ultimately contribute to improving diagnostic accuracy, optimizing personalized therapeutic strategies, and reducing the individual and socioeconomic impact of PD.

## Data Availability

All the data and supportive information are provided within the article.

## LIST OF ABBREVIATIONS

CBT: Cognitive Behavioral Therapy
CPQ: Computer Proficiency Questionnaire
HF: High Frequency
HRV: Heart Rate Variability
IPQ: Igroup Presence Questionnaire
LF: Low Frequency
LF/HF: Sympathovagal Balance
PD: Panic Disorder
pNN50: Percentage of successive NN intervals differing by more than 50 ms
RR: Respiratory Rate
rMSSD: Root Mean Square of Successive Differences
SCL: Skin Conductance Level
SDNN: Standard Deviation of NN intervals
SSQ: Simulator Sickness Questionnaire
SUDS: Subjective Units of Distress Scale
VR: Virtual Reality

## STANDARDS OF REPORTING

STROBE guidelines were followed.
